# Comparative genomics of *Staphylococcus capitis* reveals species determinants

**DOI:** 10.3389/fmicb.2022.1005949

**Published:** 2022-09-30

**Authors:** Charlotte E. Chong, Rebecca J. Bengtsson, Malcolm James Horsburgh

**Affiliations:** Institute of Infection, Veterinary and Ecological Sciences, University of Liverpool, Liverpool, United Kingdom

**Keywords:** *Staphylococcus capitis*, genome, scalp, dandruff, genomics, species determination, phenotypes

## Abstract

*Staphylococcus capitis* is primarily described as a human skin commensal but is now emergent as an opportunistic pathogen isolated from the bloodstream and prosthetic joint infections, and neonatal intensive care unit (NICU)-associated sepsis. We used comparative genomic analyses of *S. capitis* to provide new insights into commensal scalp isolates from varying skin states (healthy, dandruff lesional, and non-lesional), and to expand our current knowledge of the species populations (scalp isolates, *n* = 59; other skin isolates, *n* = 7; publicly available isolates, *n* = 120). A highly recombinogenic population structure was revealed, with genomes including the presence of a range of previously described staphylococcal virulence factors, cell wall-associated proteins, and two-component systems. Genomic differences between the two described *S. capitis* subspecies were explored, which revealed the determinants associated exclusively with each subspecies. The subspecies *ureolyticus* was distinguished from subspecies *capitis* based on the differences in antimicrobial resistance genes, β-lactam resistance genes, and β-class phenol soluble modulins and gene clusters linked to biofilm formation and survival on skin. This study will aid further research into the classification of *S. capitis* and virulence-linked phylogroups to monitor the spread and evolution of *S. capitis*.

## Introduction

*Staphylococcus capitis* was first isolated from healthy human skin in 1975 and classified as a coagulase-negative *Staphylococcus* species (CoNS) (Kloos and Schleifer, 1975). *S. capitis* is frequently found on the human scalp and the forehead and thrives in lipid-rich areas where sebaceous glands are abundant. The species was recently associated with dandruff-presenting scalps (Kloos and Schleifer, 1975; Maggs and Pennington, 1989; Bannerman and Kloos, 1991; Grimshaw et al., 2019). *S. capitis* includes two subspecies, *capitis* and *ureolyticus*, based on their differences in urease production and maltose fermentation (Bannerman and Kloos, 1991). Urease production, encoded in staphylococci by *ureDEFG*, has been reported to be crucial to bacterial adaptation, virulence, and host immune defense (Vandecandelaere et al., 2017). Although the function of urease in *S. capitis* subspecies specifically is not clear, recent studies suggest that urease is essential for pH homeostasis, viability under weak acid stress, and CoNS survival within multispecies biofilms (Bore et al., 2007; Vandecandelaere et al., 2017; Zhou et al., 2019). Therefore, urease production in *S. capitis* ssp. *ureolyticus* could aid in *S. capitis* colonization and infection.

Previous investigations sought to characterize the two *S. capitis* subspecies isolated from NICUs, with respect to clinically relevant phenotypes, including antimicrobial susceptibility, the structure of the *ica* operon, and biofilm formation (Cui et al., 2013, 2015). Studies associate increased prevalence of multidrug resistance (MDR), biofilm formation ability, and variation in the *ica* operon with *S. capitis* ssp. *ureolyticus* compared to *S. capitis* ssp. *capitis* (Cui et al., 2013), as well as differences in transcriptional response to erythromycin (Cui et al., 2015). While studies have described the *S. capitis* subspecies, none have sought to genotypically characterize them using WGS.

Many reports link *S. capitis* with a range of human diseases, being isolated frequently from prosthetic joint infections (Tevell et al., 2017, 2020; Flurin et al., 2019), prosthetic valve endocarditis (Cone et al., 2005; Nalmas et al., 2008; Al Hennawi et al., 2020), and late-onset sepsis in newborns at neonatal intensive care units (Rasigade et al., 2012; Cui et al., 2013; Carter et al., 2018; Stenmark et al., 2019; Wirth et al., 2020). The role of *S. capitis* in these infections was studied with reference to other well-described and clinically important species within the Epidermidis cluster group, i.e., *Staphylococcus epidermidis* and *Staphylococcus caprae* (Lamers et al., 2012; Cameron et al., 2015; Watanabe et al., 2018). *S. capitis* encodes important virulence factors required for biofilm production, persistence, and immune evasion (Cameron et al., 2015). The species is considered, in common with other CoNS species, such as *S. epidermidis*, to have a lower virulence potential than *S. aureus* because CoNS encode a reduced suite of exotoxins associated with invasive disease (Massey et al., 2006; Otto, 2009).

Species of CoNS do possess sufficient virulence factors to be opportunistic pathogens, which explains their contribution to the burden of nosocomial infections (Heilmann et al., 2019). Several CoNS possess a sufficiently large repertoire of virulence factor genes, including those linked to adhesion and biofilm formation, affording them both commensal and pathogenic traits (Otto, 2009; Becker et al., 2014; Heilmann et al., 2019). Important to the evolution of the genus, CoNS are proposed to act as a reservoir of mobile genetic elements (MGEs) (Heilmann et al., 2019). In their commensal life cycle, they exist closely with other bacteria on the skin and mucosal surfaces and increase their genetic diversity *via* recombination and frequent acquisition of MGE (Otto, 2013; Becker et al., 2014; Argemi et al., 2019).

In the study presented here, we whole-genome sequenced 59 *S. capitis* isolates sampled from the scalp skin and performed whole-genome sequencing analysis (WGSA), incorporating a further 127 publicly available sequences, with the aim to expand knowledge of *S. capitis* population structure, the genotypic definition of subspecies, and identify factors that are likely to contribute to virulence, competition, and colonization.

## Materials and methods

### Dataset and bacterial isolates

Whole-genome sequencing (WGS) was performed on 59 *S. capitis* isolates of scalp skin (healthy scalp site, *n* = 22; dandruff scalp site, *n* = 17; healthy site of dandruff scalp, *n* = 20) collected in the UK in 2017. Scalp samples were obtained using the method of Williamson and Kligman (1965). The collection sample site was located and an appropriate clear and straight parting in the hair (~10 cm long) was secured to maximize exposure of the scalp. A Teflon cup (18 mm internal diameter and 6 cm high) was placed onto the hair parting. A volume of 2.0 mL sampling collection medium (phosphate buffered saline plus 0.1% Triton-X-100) was applied to the collection site, and the skin was agitated in the liquid for 1 min with a Teflon rod. The resulting 4.0 mL of the sample was transferred to a sterile tube. For each scalp sample taken, 100 μL of wash was plated on agar and up to four distinct colonies were isolated from the staphylococcal selective medium: [1% (w/v) Tryptone (Oxoid), 0.5% (w/v) lab lemco powder (Oxoid), 0.3% (w/v) yeast extract, 1.3% (w/v) agar (Lab M), 0.1% (w/v) sodium pyruvate (JT Baker Chemicals), 0.05% (w/v) glycine (JT Baker Chemicals), 2.25% (w/v) potassium thiocyanate (JT Baker Chemicals), 0.12% (w/v) NaH_2_PO_4_.2H_2_0 (JT Baker Chemicals), 0.2% (w/v) lithium chloride (JT Baker Chemicals), 0.5 % (v/v) glycerol (JT Baker Chemicals), 1.0% (v/v) sodium azide (Sigma Aldrich), and 3.0% (v/v) sterile egg yolk emulsion (Lab M), pH 7.2].

Additionally, seven skin isolates were sequenced that included culture collection type strains of both *S. capitis* subspecies [ATCC 49325 (*S. capitis* subsp. *ureolyticus*) and ATCC 27840 (*S. capitis* subsp. *capitis*)] (Supplementary Table 1). Also included were published genomic data available from GenBank (https://www.ncbi.nlm.nih.gov/genbank/), Sequence Read Archive (https://www.ncbi.nlm.nih.gov/sra), or European Nucleotide Archive (https://www.ebi.ac.uk/ena), and accession numbers are listed in Supplementary Table 1. A total of 11 publicly available, complete, published, and taxonomically classified as *S. capitis* genomes were downloaded. All publicly available *S. capitis* genome reads were also downloaded and subject to quality control. This study included WGS reads from Illumina sequencing only, with more than 10X coverage. All publicly available datasets were then phylogenetically reconstructed (as described below), and Treemmer v0.3 was used to reduce redundancies within the public dataset, leaving 120 genomes to be included in further analyses (Menardo et al., 2018).

### Genome sequencing and phylogenetic analysis

All isolates selectively obtained in this study, together with seven further strains comprising culture collection type strains that were included within a formally defined taxonomy (included in this study: NCTC 11045 and DSM 6717) and skin isolates, were grown in 10 mL of BHI broth (Lab M) overnight by shaking at 37 °C. Subsequently, 1 mL of each overnight culture was centrifuged for 1 min at 5,000 rpm and resuspended in 180 μl of lysis buffer (20 mM Tris-HCl pH 8, 2 mM EDTA, and 1.2% Triton-X-100); the cells of each clone were extracted to obtain high-quality genomic DNA using the QIAGEN DNeasy Blood and Tissue Kit and eluted in 10 mM Tris-HCl (pH 8.5).

The DNA concentration was measured using a Thermo Scientific Nanodrop, a Qubit plus visualization after gel electrophoresis on 1% (w/v) agarose gels (at 90 mV for 40 min with a 1 kb ladder). For sequencing, the extracted DNA in a final volume of 60 μl (concentration 1–30 ng μl^−1^) was submitted for Illumina DNA sequencing by MicrobesNG [http://www.microbesng.uk, which is supported by BBSRC (grant number BB/L024209/1)] using Nextera XT library protocol on a HiSeq platform, generating 250 bp paired-end reads (Illumina, San Diego, CA, USA). The resulting datasets are available from the SRA under BioProject number PRJEB47273. Adapters and low-quality bases were trimmed with Trimmomatic v??? (Bolger et al., 2014), and read qualities were assessed using FastQC v0.11.7 (https://www.bioinformatics.babraham.ac.uk/projects/fastqc/) and MultiQC v1.0 (Ewels et al., 2016). Genome sequences were *de novo* assembled and annotated using Unicycler v 0.4.7 (Wick et al., 2017) with default parameters, using SPAdes v 3.15.4 (Prjibelski et al., 2020) and Prokka v 1.14.6 (Seemann, 2014).

Sequence reads from 186 *S. capitis* isolates (scalp isolates, *n* = 59; other skin isolates, *n* = 7; publicly available isolates, *n* = 120) were mapped to the reference genome *S. capitis* AYP1020, which was isolated from human blood (Cameron et al., 2015) (NCBI Genome accession GCA_001028645.1) using Snippy v.4.4.3 with minimum coverage of 4 to generate core genome SNP alignment files (https://github.com/tseemann/snippy) (Cameron et al., 2015). The phylogeny of the strains was reconstructed by generating a maximum-likelihood (ML) tree with the substitution model GTR+G+ASC and 1,000 bootstrap replicates using IQTREE v 1.6.12 (Chernomor et al., 2016), based on the core genome alignment without the recombining regions identified by Gubbins v2.3.4 (Croucher et al., 2014). Gubbins was run with default parameters on the core genome alignment of 186 strains, which generated a chromosomal SNP alignment length of 59,972 bp. Additionally, regions containing phage were identified using PHASTER (Arndt et al., 2016), MGE sequences were identified from the reference genomes, and co-ordinates were used to mask these sites using BEDTools v 2.29.2 (Quinlan and Hall, 2010). The population structure was investigated using hierarchical Bayesian analysis of population structure with r-hierBAPS, specifying two cluster levels, 20 initial clusters, and infinite extra rounds (Cheng et al., 2013). Visualizations were performed using iTOL v4.2 (Letunic and Bork, 2016).

To measure the extent of *S. capitis* genomic diversity, pairwise SNP distance was determined from the alignment of core genome SNPs identified outside the regions of recombination using snp-dists v 0.7.0 (https://github.com/tseemann/snp-dists). To examine the phenotypic basis for the separation of the two subspecies within the phylogenetic reconstruction (Figures 2, 3), the API *Staph-Ident* Strip system was used to analyse the biochemical profiles of multiple isolates included in this study (BioMérieux, Marcy l'Etoile, France). The API Staph-Ident Strip system was used according to the manufacturer's instructions.

### Pan-genome analysis

Pan-genome analysis of all 186 isolates was performed using the Panaroo v1.1.2 software package with default parameters, MAFFT alignment, and a core gene threshold of 90% (Tonkin-Hill et al., 2020). Predicted coding gene sequences in all isolates were extracted from the gene presence and absence matrix provided by Panaroo v.1.1.2, separated into core and accessory gene groups, and input into eggnog-mapper v 2.1.6 to identify the cluster of orthologous groups (COGs) (Huerta-Cepas et al., 2017; Tonkin-Hill et al., 2020).

To identify the genes enriched in *S. capitis* ssp. *ureolytics*, the output files from Panaroo were used as an input for Scoary v1.6.16 (Brynildsrud et al., 2016), a microbial pan-GWAS tool, to infer genes overrepresented in the subspecies. Scoary was used with the settings -no_pairwise flag, and only genes with a Benjamini–Hochberg *p* < 0.05 and an odds ratio (OR) > 1 were considered to be overrepresented in the subspecies cluster.

### *In silico* analysis of dataset

Genetic determinants for AMR were identified using ABRicate v0.9.8 (https://github.com/tseemann/abricate) with NCBI Bacterial Antimicrobial Resistance Reference Gene Database, with a default minimum DNA percentage identity of 80% (Feldgarden et al., 2019). Other potential virulence factors, such as phenol soluble modulins and exoenzymes, cell wall-associated proteins, and two-component systems were identified by homology searches, using BLASTp of annotated reference genomes (*S. capitis* AYP1020 (Genbank assembly accession: GCA_001028645.1), *S. epidermidis* RP62a (Genbank assembly accession: GCA_000011925.1), and *S. aureus* MW2 (Genbank assembly accession: GCA_00307695.1) and pan-genome data from various studies (Altschul et al., 1990; Baba et al., 2002, 2008; Gill Steven et al., 2005; Cameron et al., 2015).

Average nucleotide identity (ANI) indices were used to quantify the genetic relatedness of the two *S. capitis* subspecies. ANI estimates the genetic relatedness between two genomes to assess species boundaries. To compare the genetic relatedness of the *S. capitis* subspecies in this study, FastANI v1.2 was used with default settings to compare all isolates of potential ssp. *ureolyticus* and ssp. *capitis* isolates, using the recommended cut-off score of >95% that indicates isolates belong to the same species (Jain et al., 2018). *S. capitis* ssp. *ureolyticus* culture collection isolates were also compared to other ssp. *ureolyticus* isolates, similar to Bannerman and Kloos (1991).

### Ethics approval

Written informed consent was obtained from all enrolled participants. The study protocol was reviewed and approved by an Independent Ethics Committee and operated at Unilever Port Sunlight, United Kingdom. The study was conducted in compliance with the Declaration of Helsinki and its subsequent revisions.

## Results

### Genome composition

*Staphylococcus capitis* sequence reads (scalp isolates, *n* = 59, other skin isolates, *n* = 7, publicly available isolates, *n* = 120) were assembled into draft genomes with an average of 85 contigs. The mean size of the assembled genomes ranged from 2.2 to 2.6 Mb. Each genome had a mean 2,335 (2,087–2,565) predicted protein sequences (CDSs) with a meanGC content of 32.7%, similar to the *S. capitis* reference genome AYP1020 (Cameron et al., 2015).

**Figure 1:**
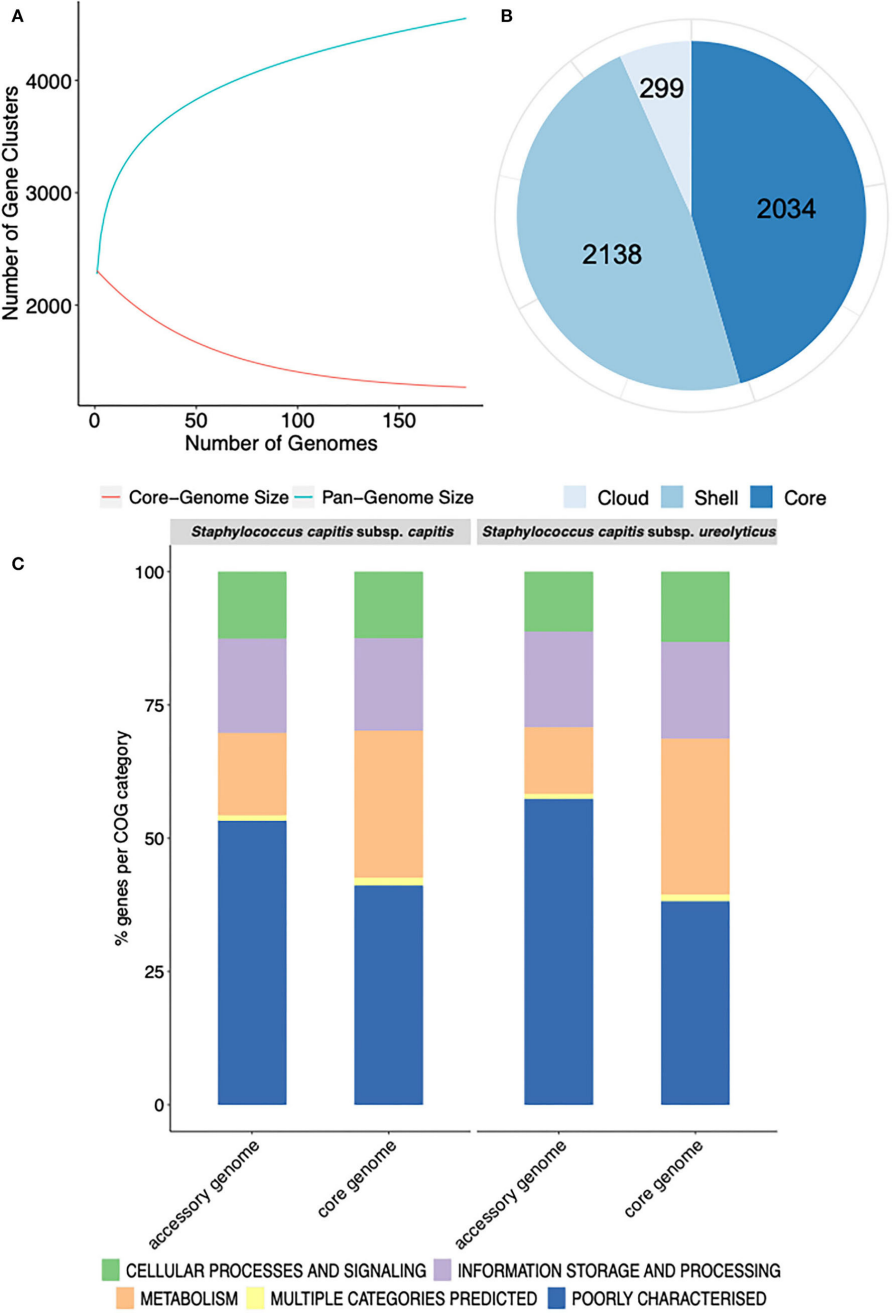
Representation of the pan-genome and COG functional annotation of *S. capitis* genomes. **(A)** Pan-genome curve generated by plotting the total number of gene families in the pan (blue) and core (red) genome of *S. capitis*. The graph represents how the pan- and core-genomes vary as genomes are added in random order. As the number of genomes increased, the pan-genome increased. **(B)** *Staphylococcus capitis* pan-genome statistics. The size of the pan-genome, including core (shared by >95% of isolates), shell (found in 15–95% of isolates), and cloud (found in <15% of isolates) genes. **(C)** Functional annotation of the core and accessory genomes of *S. capitis* subsp. *capitis* and *S. capitis* subsp. *ureolyticus*. Percentages of the core and accessory genomes annotated according to COG functional categories.

The pan-genome of the 186 *S. capitis* isolate dataset comprised 4,471 unique clusters of orthologous groups (COGs). The pan-genome was further divided into 2,034 (45.4%) core genes (shared by all genomes) and 2,437 (54.5%) accessory genes (shared by some genomes) (Figure 1B). Gene accumulation curves reflected an open pan-genome, where the addition of each new genome increases the total gene pool (Figure 1A). *S. capitis*, like other CoNS, has a somewhat limited core genome but displays an open pan-genome due to the introduction of novel or accessory genes by means of HGT.

Further pan-genome analysis of each *S. capitis* subspecies revealed that most annotated genes in the accessory genomes of both *S. capitis* ssp. *capitis* (53 %) and ssp. *ureolyticus* (57 %) were poorly characterized, which was also true of the core genome (ssp. *capitis* 41 % and ssp. *ureolyticus* 38 %). The finding could indicate the presence of novel gene clusters in the *S. capitis* genome and a low level of curation. The most abundant categories in the core genomes of both subspecies were those linked to essential gene functions, such as transcription (ssp. *capitis* 7 % and ssp. *ureolyticus* 7 %). In contrast, the accessory genomes were enriched with gene clusters associated with replication, recombination, and repair (ssp. *capitis* 9 % and ssp. *ureolyticus* 9 %), as observed in the pan-genome analysis of each subspecies (Figure 1C).

### Population structure and genetic diversity

**Figure 2:**
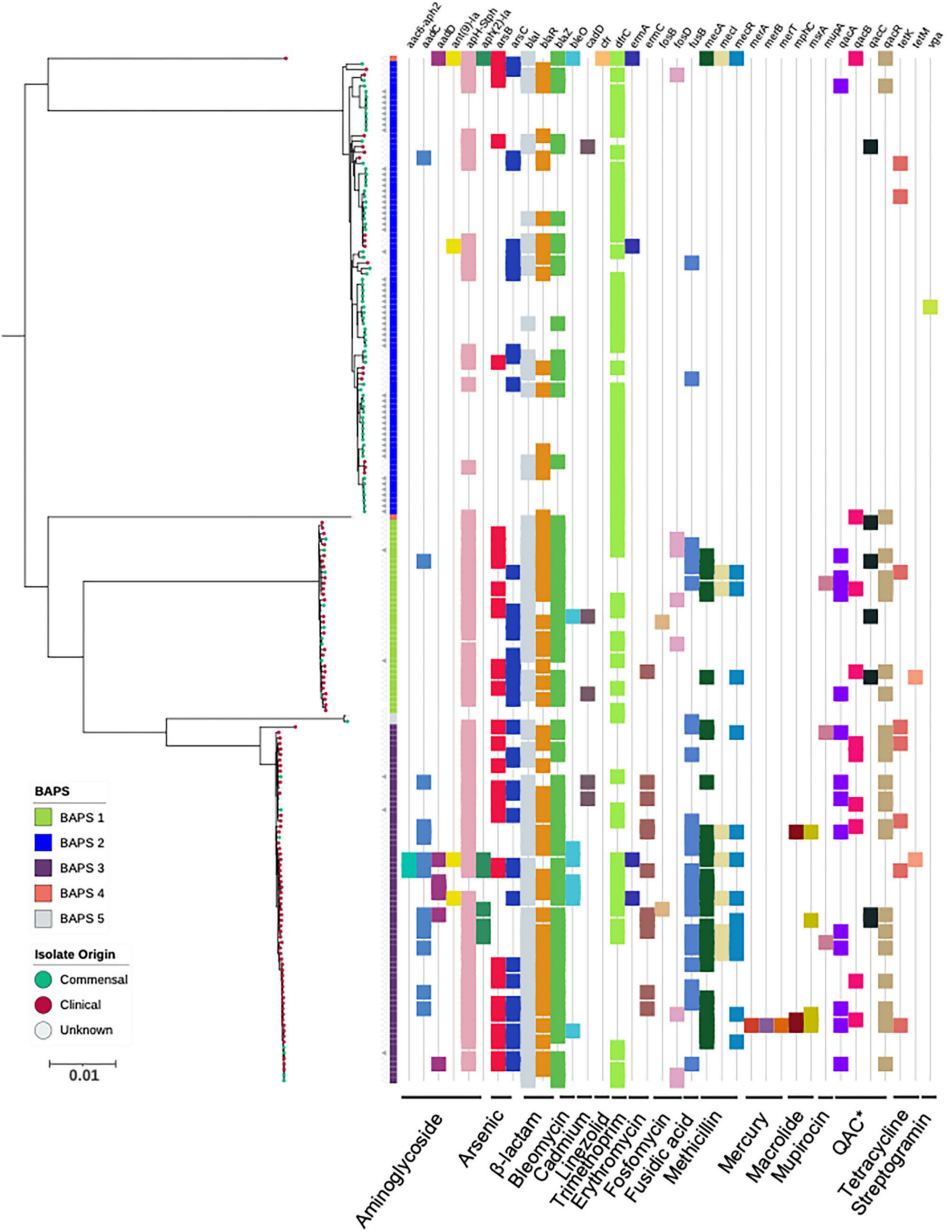
Maximum-likelihood phylogeny based on core genome alignments of 186 *S. capitis* isolates, presenting the presence and absence of antimicrobial resistance genes. ML tree is midpoint rooted, and bootstrap support values were calculated from 1,000 replicates. The first color block represents rhierBAPS clustering, dots describe the setting where isolates were retrieved; green = commensal (including scalp samples from this study), red = clinical, and gray = unknown. Filled gray triangles describe scalp isolates from this study. The subspecies differentiation of *S. capitis* is presented as the subclades described as BAPS groups 1, 3, 4, and 5. The presence (colored blocks) and absence (white blocks) of antimicrobial resistance is denoted for each isolate (*Quaternary Ammonium Compounds). The scale bar represents the number of nucleotide substitutions per site. The figure was visualized using iTol v 4.2 (Letunic and Bork, 2016).

To explore the population structure of *S. capitis*, a maximum-likelihood phylogenetic tree was constructed based on a chromosomal SNP alignment length of 59,972 bp. This revealed two distinct clades separated by an average pairwise distance of 7,538 core genome SNPs. The position of strains included in this study to contextualize scalp isolates within an established *S. capitis* population and described in the literature as either *S. capitis* subspecies, as well as culture collection type strains within the phylogenetic reconstruction, was used to determine that *S. capitis* ssp. *capitis* isolates belonged to the upper clade and ssp. *ureolyticus* to the bottom clade (Figure 2). While the *S. capitis* ssp. *capitis* clade comprised a single dominant subclade, *S. capitis* ssp. *ureolyticus* is more diverse and comprises three clades. Population structure was also inferred using BAPS to cluster genomes based on shared patterns of variation, which was congruent with the phylogeny. In this study, clinical isolates are defined as those isolated from a clinical setting, e.g., a hospital neonatal unit, or from a host with a disease state, whereas commensal isolates are defined as those isolated from healthy hosts and the community. Isolate origins were overlaid onto the phylogeny and revealed that clinical isolates were predominantly found in the *S. capitis* ssp. *ureolyticus* clade (78/106), while commensal isolates were associated with the *S. capitis* ssp. *capitis* clade (59/82). Of note, 22 clinical isolates are interspersed across the dominant subclade within the *S. capitis* ssp. *capitis* clade and 23 commensal isolates are interspersed across the two *S. capitis* ssp. *ureolyticus* subclades (Figure 2). The distribution could indicate that commensal and clinical isolates belonging to each subspecies are genetically similar and evolved from a common ancestor or alternatively *S. capitis* is a true opportunistic pathogen, and many strains have disease potential.

Extensive recombination was observed among the study isolates, with recombination most evident in BAPS clusters 2, 3, 4, and 5, which collectively contain 293 recombination blocks. BAPS cluster 1 revealed less recombination (a total of two recombination blocks); however, this is likely due to the reference genome itself being clustered in this group. Recombination was inferred across large regions of the chromosome, predominantly within the first ~750 kb and the last ~1 Mb of the genome (Supplementary Figure 1). The recombination data are consistent with *S. capitis* having arisen following extensive recombination events (Carter et al., 2018).

### Insights into *S. capitis* pathogenicity

The CoNS that colonize human skin are generally considered to be non-pathogenic species specialized for healthy human skin and mucosal surfaces, but they are now emerging as important opportunistic pathogens (Otto, 2009, 2013; Post et al., 2017; Méric et al., 2018; Espadinha et al., 2019; Heilmann et al., 2019). Antimicrobial resistance properties are important factors of nosocomial infections. Therefore, we screened for the presence of genetic determinants for AMR and identified genes predicted to encode for resistance against tetracyline, β-lactam, bleomycin, fosfomycin, methicillin resistance, fusidic acid streptogramin A, macrolide, linezolid, trimethoprim, and aminoglycoside. Amongst the *S. capitis* genomes analyzed, 48% (5% *S. capitis* ssp. *capitis* and 44% *S. capitis* ssp. *ureolyticus*) were classified as MDR, carrying genetic determinants conferring resistance against three or more classes of drugs (Figure 2). MDR was found in 78% of isolates from the *ureolyticus* clade, compared to 11% of isolates from the *capitis* clade. When the dataset was stratified by isolate origin, we found clinical isolates carried more AMR genes in comparison with commensal isolates. A total of 635 AMR-linked genes were found across clinical isolates (*n* = 99) compared to 169 found in commensal isolates (*n* = 81).

**Figure 3:**
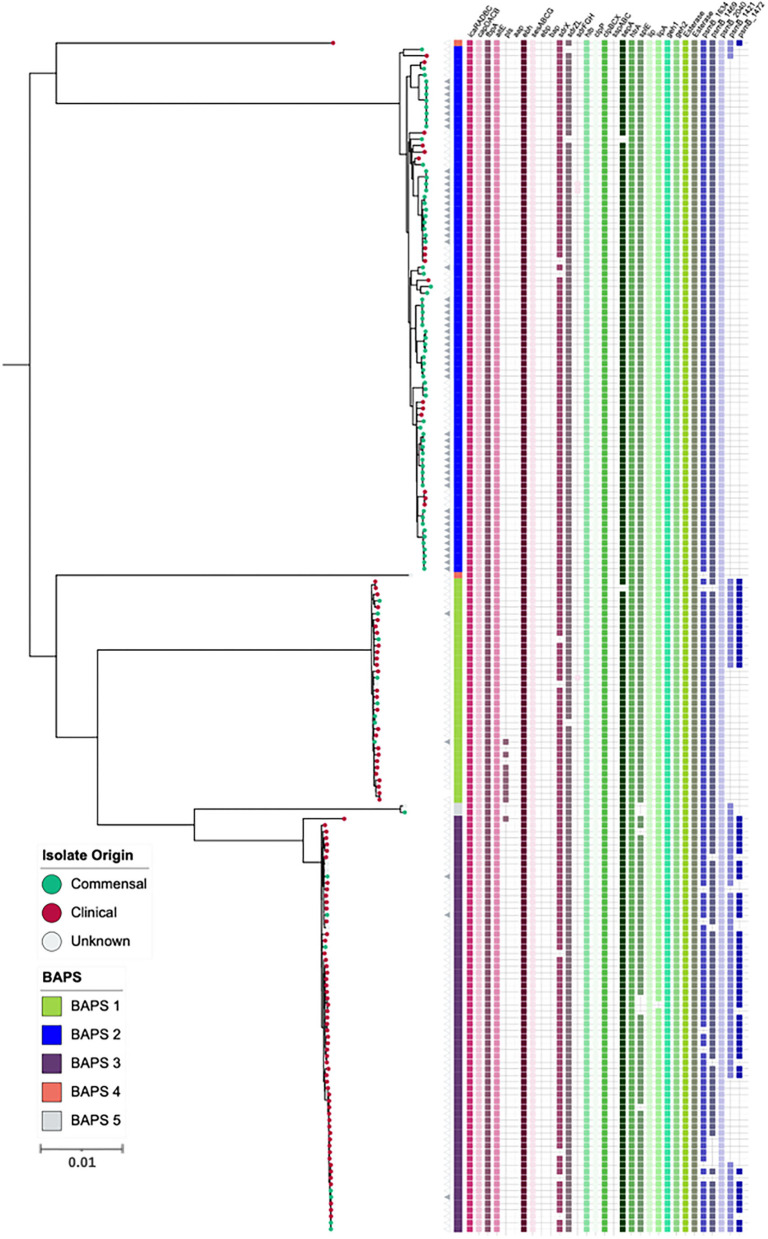
Maximum-likelihood phylogeny based on core genome alignments of 186 *S. capitis* isolates, presenting the presence and absence of genes linked to CoNS virulence potential. ML tree is midpoint rooted, and bootstrap support values were calculated from 1,000 replicates. The first color block represents rhierBAPS clustering, dots describe the setting where isolates were retrieved; green = commensal (including scalp samples from this study), red = clinical, and gray = unknown. Filled gray triangles describe scalp isolates from this study. The subspecies differentiation of *S. capitis* is presented as the subclades described as BAPS groups 1, 3, 4, and 5. The presence (colored blocks) and absence (white blocks) of virulence genes is denoted for each isolate. The scale bar represents the number of nucleotide substitutions per site. The figure was visualized using iTol v 4.2 (Letunic and Bork, 2016).

**Figure 4:**
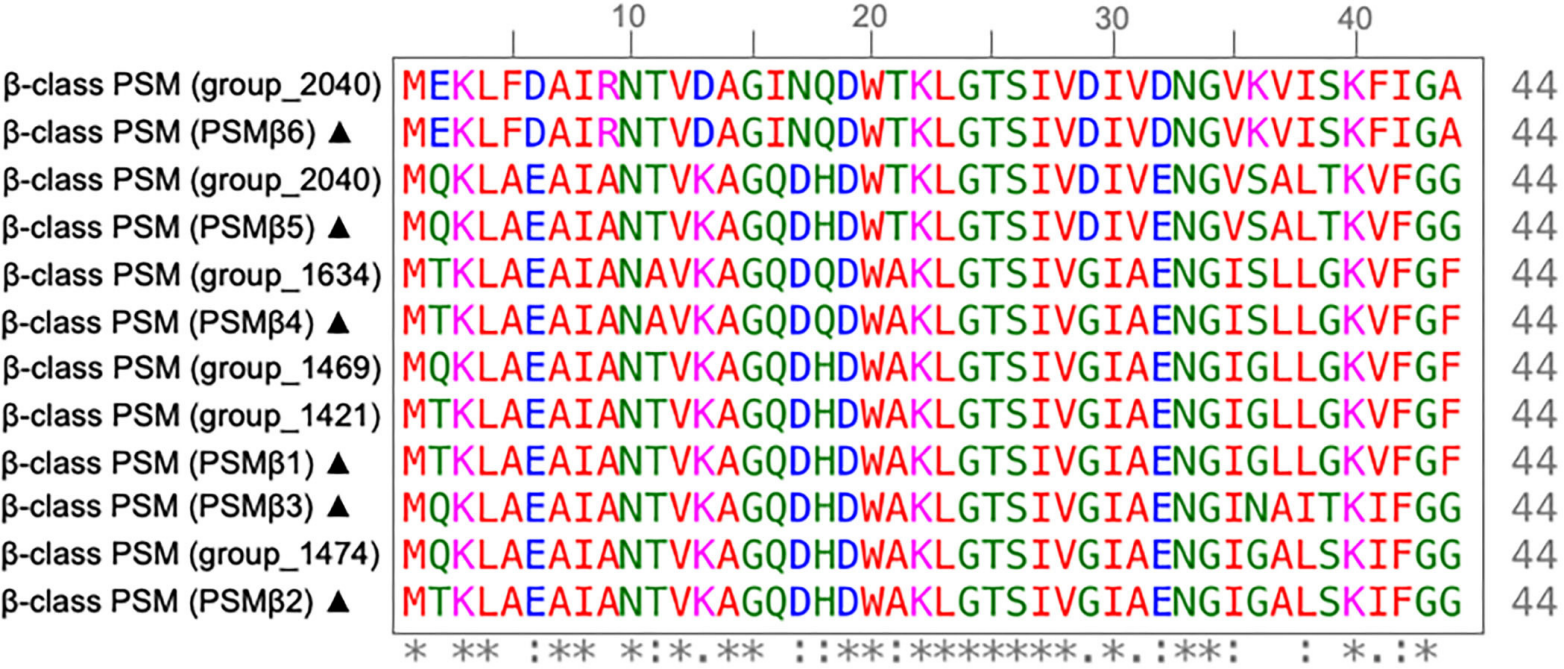
Multiple sequence alignment of β-class phenol soluble modulins (PSMs) of *S. capitis* isolates. MSA of β-class PSMs protein sequences found in *S. capitis* genomes from this study and those described by O'Neill (2020) (sequences marked with ▴) created with ClustalW (Clustal et al., 1994). Residues are colored based on an amino acid property (red: small and hydrophobic, blue: acidic, magenta: basic, green: hydroxyl, sulfhydryl, and amine, and gray: unusual), positions that contain fully conserved residues are marked with an asterisk, and positions marked with a colon indicate conservation between groups of amino acids with similar properties.

In addition to AMR genes, we also investigated the role of phenol-souble modulins (PSMs) contributing to the virulence potential of *S. capitis*. PSMs are a novel toxin family that have antimicrobial activity (Peschel and Otto, 2013; Cheung et al., 2014) and have been attributed to the competitive success of CoNS due to their ability to inhibit the growth of other commensal bacteria, such as *Cutibacterium acnes* (O'Neill, 2020). A total of five gene clusters encoding β-class PSMs were identified (Figure 3), with gene clusters 1,634, 1,469, and 2,040 found in >98% of isolates, sharing >90 % similarity when locally aligned to PSMs described and isolated from *S. capitis*, by O'Neill (2020). Similar to AMR gene presence and absence, PSM-associated gene clusters were found more abundantly in the *S. capitis* ssp. *ureolyticus* clade relative to the *S. capitis* ssp. *capitis* clade (Figure 3). Specifically, gene clusters 1,421 and 1,472 were found in 66 and 55% of isolates from the *ureolyticus* clade, in contrast to 3 and 0% of isolates from the *capitis* clade (Figure 4).

To further investigate variation in the β-class PSMs identified, we performed multiple sequence alignment of corresponding amino acid sequence from the six gene clusters, which revealed conservation of residues attributed to the maintenance of the amphipathic nature of the peptides, essential to PSM antimicrobial activity (Kumar et al., 2017). Specifically, lysine at the 3^rd^ and/or tryptophan at the 20^th^ position are putatively associated with providing antibacterial activity of β-class family peptides (Kumar et al., 2017), both of which are conserved in the peptide sequences of this study (Figure 4).

Additionally, we specifically screened for the presence of orthologous CDS that likely contribute to *S. capitis* pathogenicity, including staphylococcal cell wall-associated (CWA) proteins curated with potential virulence roles; biofilm-associated proteins IcaRADBC; capsule biosynthesis proteins CapDACB; surface adhesins AtlE, Pls, Aap, FnbpA, SesA, SesB, SesC, SesG, Ebp, and Bap; and MSCRAMMs SdrX, SdrZL, SdrH, SdrF, and SdrG. Of these CWA proteins, 11 were encoded in *S. capitis* AYP1020. Across the 186 *S. capitis* genomes analyzed, *sesA, sesB, sesC, sesG, icaRADBC, fbnpA, capDACB*, and *atlE* were found in all isolates. MSCRAMM genes *sdrX* and *sdrZL* were found in >95 % of genomes investigated. Contrastingly, the surface adhesin gene *pls* was found in <10 % of *S. capitis* genomes and was absent from *ssp. capitis* (Figure 3). Notably, genes that were absent from the *S. capitis* AYP1020 genome (determined in this study as *S. capitis* ssp. *ureolyticus*) but present in other CoNS species, such as *S. epidermidis* RP62a (Cameron et al., 2015), included *sdrF, ebp, bap, sdrH, sdrG*, and *aap*. These genes were absent in >95 % of the genomes included in this study (Cameron et al., 2015) (Figure 3). The secreted protein genes *hlb, clpP, clpBCX, sepA, htrA, lip, geh1, geh2*, and *lipA* were present in >95 % of all the isolates. The presence of a suite of exoproteins could contribute to host colonization, persistence, infection, and immune evasion, important to both pathogenesis and colonization (Heilmann et al., 2019). Notably, *sspA, sspB*, and *sspC* were absent in *S. capitis* genomes; the serine protease SspA promotes invasion in *S. aureus* (Dubin et al., 2001) (Figure 3).

The 16 two-component systems (TCS) described in *S. aureus* are conserved in other closely related CoNS, indicative of adaptive and highly versatile species (Haag and Bagnoli, 2017; Rapun-Araiz et al., 2020). The number of TCS found within bacterial genomes is proportional to genome size, diversity of environment, and the complexity of bacterial cellular processes (Rapun-Araiz et al., 2020). TCS form part of complex regulatory systems that respond to multiple environmental signals and are vital to the capacity of staphylococcal species to colonize, survive on different body surfaces, and cause a diverse spectrum of diseases (Haag and Bagnoli, 2017; Coates-Brown et al., 2018). *S. aureus* TCS are extensively characterized, and therefore the 186 *S. capitis* genomes included in this study were screened for homologous protein sequences. Of the 16 TCS, 14 were found in *S. capitis*. This is intermediate with *S. epidermidis* (16 TCS) and *S. saprophyticus* (11 TCS) with numbers determined by Rapun-Araiz et al. (2020) to be indeterminate of genome size in staphylococci (Table 1).

**Table 1:** Two-component systems in *S. aureus* and *S. capitis*.

**Two-component system**	***S. aureus* reference**	***S. capitis* reference**	**Presence in *S. capitis* ssp. *capitis* (%)**	**Presence in *S. capitis* ssp. *ureolyticus* (%)**	**Function**
*walRK*	MW0018	AYP1020_RS09955	100	100	Cell wall maintenance, cell viability
	MW0019	AYP1020_RS09960	100	100	
*hptSR*	MW0198		0	0	Intracellular survival, uptake of hexose phosphate
	MW0199		0	0	
*lytSR*	MW0236		0	0	Autolysis, eDNA release, biofilm
	MW0237		0	0	
*graRS*	MW0621	AYP1020_RS00130	100	100	AMPs resistance, growth at low pH
	MW0622	AYP1020_RS00135	100	100	
*saeSR*	MW0667	AYP1020_RS00365	100	100	Virulence factors regulation
	MW0668	AYP1020_RS00360	100	100	
*tcs7SR*	MW1208	AYP1020_RS03075	100	100	Uncharacterised function
	MW1209	AYP1020_RS03080	100	100	
*arlRS*	MW1304	AYP1020_RS03545	100	100	Pathogenesis mechanisms
	MW1305	AYP1020_RS03540	100	100	
*srrBA*	MW1445	AYP1020_RS03875	100	100	Anaerobic respiration, metabolism, growth at low temperatures
	MW1446	AYP1020_RS03880	100	100	
*phoRP*	MW1636	AYP1020_RS04760	100	100	Phosphate uptake and homeostasis
	MW1637	AYP1020_RS04765	100	100	
*airRS*	MW1789	AYP1020_RS05370	100	100	Oxidative stress response
	MW1790	AYP1020_RS05375	100	100	
*varRS*	MW1824	AYP1020_RS05700	100	100	Cell wall-affecting antibiotic resistance, cell wall biosynthesis
	MW1825	AYP1020_RS05705	100	100	
*agrCA*	MW1962	AYP1020_RS06010	100	100	Quorom sensing control of adhesion and virulence factors
	MW1963	AYP1020_RS06005	100	100	
*kpdDE*	MW2002	AYP1020_RS09655	100	100	Potassium homeostasis regulation
	MW2003	AYP1020_RS09660	100	100	
*hssRS*	MW2282	AYP1020_RS07580	100	100	Heme metabolism regulation
	MW2283	AYP1020_RS07585	100	100	
*nreCB*	MW2313	AYP1020_RS07750	100	100	Response to low oxygen, nitrate reduction
	MW2314	AYP1020_RS07755	100	100	
*braSR*	MW2544	AYP1020_RS08920	100	100	Antimicrobial peptide resistance
	MW2545	AYP1020_RS08925	100	100	

Presence and absence of the 16 TCS of *S. aureus* (MW2) described in *S. capitis* reference genome AYP1020 and isolates included in this study.

### *S. capitis* subspecies definition

To biochemically assess the differences between the two subspecies *S. capitis* ssp. *capitis* and *S. capitis* ssp. *ureolyticus* based upon the original descriptions of Bannerman and Kloos (1991), the API *Staph-Ident* Strip system was used (BioMérieux, Marcy l'Etoile, France). A total of 22 isolates sampled in this study that were spread across the phylogenetic tree (11 assigned to the *S. capitis* ssp. *capitis* clade and 11 assigned to the *S. capitis* ssp. *ureolyticus* clade), as well as the type and culture collection stains, were tested for classifying the phenotypic traits. Among the 11 isolates belonging to the *S. capitis* ssp. *ureolyticus* clade, only 4 (33%) tested urease-positive/maltose-positive, and 10 (91%) *S. capitis* ssp. *capitis* isolates tested urease-negative/maltose-negative, indicating an unreliable phenotype of urease activity and maltose fermentation as a subspecies definition (Supplementary Figure 2).

Since the original phenotypic trait descriptors for *S. capitis* did not sufficiently discriminate between the subspecies, we then investigated the correlation between the presence of genes *ureDEFG*, which are known to control urease production in staphylococci (Vandecandelaere et al., 2017). These genes were found to be a part of the *S. capitis* core genome from the pan-genome analysis. However, as urease production is inducible and controlled by a complex network of genes, including CcpA, Agr, and CodY in *S. aureus* (Zhou et al., 2019), we sought to quantify and analyse the genetic differences between *S. capitis* ssp. *ureolyticus* and capitis. Analysis of the average nucleotide identity (ANI) between isolates determined here as *S. capitis* ssp. *ureolyticus* and *S. capitis* ssp. *capitis* revealed that genomes from the two subspecies shared little genetic differences with 96% ANI. Pan-genomic comparative analysis also revealed limited gene content variation of 1% between the two subspecies. To help identify genes that could be used to discriminate between the subspecies and serve as diagnostic markers for rapid identification by PCR in future studies, we identified significantly overrepresented genes of each subspecies. This approach identified a total of 38 gene clusters found across all *S. capitis* ssp. *capitis* genomes and 13 across all *S. capitis* ssp. *ureolyticus* genomes (Table 2, Supplementary Tables 2, 3). Upon closer inspection of differential gene output from Scoary, the majority of unique genes identified were in fact divergent gene orthologs (Table 2, Supplementary Table 2). An example of this is the *icaC* gene, which encodes for an intercellular adhesion protein. While one version of this gene cluster was only found in *S. capitis* ssp. *ureolyticus* isolates (98%), another version sharing blastp identity of 22% was only present in *S. capitis* ssp. *capitis* isolates (0%).

**Table 2:** Gene clusters found significantly enriched in either *S. capitis* ssp. *capitis* or ssp. *ureolyticus* (*p* < 0.001).

**Gene name**	***S. capitis* reference**	**Annotation**	***S. capitis* ssp. *capitis* (%)**	***S. capitis* ssp. *ureolyticus* (%)**
*group_2869*		Hypothetical protein	100	0
*group_459*		Putative phage head morphogenesis protein	100	0
*crtNX*		Dehydrosqualene desaturase	100	4.76
*hdcA*		Histidine decarboxylase proenzyme	100	6.67
*hsdM*		Type I restriction-modification system methyltransferase subunit	100	21.90
*group_1023*		Putative transcriptional regulator	100	29.52
*group_2665*	AYP1020_RS09385	Putative protein YjdF	100	34.29
*sasCX*		Cell-wall-anchored protein SasC	100	35.24
*arcAX*		Arginine deiminase	98.72	6.67
*arcCX*		Carbamate kinase ArcC1	98.72	7.62
*arcDX*		Arginine/ornithine APC family amino acid-polyamine-organocation transporter antiporter	98.72	7.62
*argFX*		Ornithine carbamoyltransferase	98.72	7.62
*hsdSX*		Type I restriction modification DNA specificity protein;type I restriction modification system site specificity determination subunit HsdS_1	98.72	7.62
*trkG*		Trk family potassium (K+) transporter ABC protein	98.72	11.43
*arsB*		Arsenical pump membrane protein	98.72	16.19
*cdr*		Coenzyme A disulfide reductase	98.72	19.05
*arsR*		Arsenical resistance operon repressor	98.72	19.05
*group_2407*		Arsenate reductase (glutaredoxin)	98.72	20.95
*arsA*		Arsenical pump-driving ATPase	98.72	55.24
*arsD*		Arsenical resistance operon trans-acting repressor ArsD	92.31	37.14
*pyrBX*		Aspartate carbamoyl transferase catalytic subunit	98.72	0.95
*group_1353*	AYP1020_RS02690	Hypothetical protein	0	100
*group_798*	AYP1020_RS11575	Hypothetical protein	0	100
*group_1413*	AYP1020_RS11570	Hypothetical protein	0	99.05
*icaCX*	AYP1020_RS08445	Putative poly-beta-16-N-acetyl-D-glucosamine export protein	0	98.10
*fmrO*	AYP1020_RS12300	rRNA methyltransferase FmrO; hypothetical protein	35.90	96.19
*dapF*	AYP1020_RS09635	Diaminopimelate epimerase	35.90	96.19
*qorB*	AYP1020_RS06480	Cobalt (Co2+) ABC superfamily ATP binding cassette transporter membrane protein	0	78.10
*group_1472*		Antibacterial protein (phenol soluble modulin)	0	55.24
*mqo*		Putative malate: quinone oxidoreductase 2	0	64.76
*pfpI*		Intracellular protease	0	60
*opp1B*		Oligopeptide ABC superfamily ATP binding cassette transporter membrane protein	35.90	84.76
*opp1D*		Oligopeptide ABC superfamily ATP binding cassette transporter ABC protein	35.90	84.76
*group_687*		Hypothetical protein	35.90	84.76
*group_585*		MFS family major facilitator transporter	35.90	84.76
*opp1A*		Oligopeptide ABC superfamily ATP binding cassette transporter binding protein	35.90	84.76
*opp1C*		Oligopeptide ABC superfamily ATP binding cassette transporter membrane protein	35.90	84.76
*opp1F*		ABC superfamily ATP binding cassette transporter ABC protein	35.90	84.76
*blaRX*		β-lactamase regulatory protein	6.41	62.86

Gene clusters, presence and absence, and functional descriptions were obtained from Panaroo and Scoary pangenome analysis of assembled genomes. X these genes exist in different conserved versions in isolates. *S. capitis* reference gene numbers are from *S. capitis* AYP1020 (Genbank Assembly Accession: GCA_001028645.1) (Cameron et al., 2015). The complete list can be found at Supplementary Table 2.

To further investigate specific genetic signatures associated with each *S. capitis* subspecies, we applied Scoary to identify genes that are overrepresented in *S. capitis* ssp. *capitis* and *S. capitis* ssp. *ureolyticus*. A total of 1,086 predicted gene clusters were found to differ significantly (*p* < 0.05) between the subspecies, although no significant differences were found between the assigned functional COG categories (Table 1, Supplementary Table 2, Supplementary Figure 1). Gene clusters with a known function identified as being enriched in *S. capitis* ssp. *capitis* isolates include those for the arginine catabolic mobile element (ACME), encoding arginine deiminase activity found in various species of *Staphylococcus*. Most *S. capitis* isolates in this study contain a Type V ACME gene cluster; however, different conserved versions are found in each subspecies. ACME types are currently characterized by the presence and absence of the opp3 operon, encoding an arginine deaminase pathway, the *arc* operon, an oligopeptide permease ABC transporter, and *kdp* operon, encoding a potassium transporter (Diep et al., 2008; Planet et al., 2013; Lindgren et al., 2014; O'Connor et al., 2018). A Type V ACME gene cluster is indicated by the presence of all three associated operons (O'Connor et al., 2018). Additional gene clusters enriched in ssp. *capitis* included those encoding SasC cell wall anchored protein and CrtN dehydrosqualene desaturase involved in staphyloxanthin biosynthesis (Table 2, Supplementary Tables 2, 3). In *S. capitis* ssp. *ureolyticus*, genes predicted to encode for virulence factors were found to be enriched, specifically genes with antimicrobial-associated functions, including β-lactam resistance protein BlaR and β-class phenol soluble modulins (Table 2, Supplementary Tables 2, 3). This is concurrent with the current literature that *S. capitis* ssp. *ureolyticus* as the more virulent subspecies (Cui et al., 2013; Tevell et al., 2020).

## Discussion

*Staphylococcus capitis* is an opportunistic pathogen that is associated with increasing reports of bloodstream infections and neonatal infections in intensive care units. Currently, *S. capitis* is mostly studied with reference to the well-described and clinically important *S. epidermidis*. In the absence of a prior expansive study of *S. capitis* genomes from the scalp, the current work aimed to explore the WGS of the species. The aim was to expand knowledge of its population structure and compare genomic differences between commensal and clinical isolates to gain an understanding of the genetic factors that contribute to *S. capitis* pathogenicity.

Pan-genome analysis indicated that the *S. capitis* has an open pan-genome, which could arise from a capacity to acquire exogenous DNA while living in extensive bacterial communities. The large accessory genome size suggests that *S. capitis* contains a large repertoire of genes that confer advantages under particular environmental conditions to support its colonization and/or cause infections in clinical settings. Similarly, other members of the Epidermidis cluster group, such as *S. caprae* and *S. epidermidis*, have a large, open pan-genome state that contrasts with certain other CoNS, e.g., *Staphylococcus lugdunensis* (Argemi et al., 2018; Sun et al., 2020). It can therefore be hypothesized that in common with other staphylococci, horizontal gene transfer (HGT) has led to the acquisition of virulence genes within *S. capitis* genomes (Sun et al., 2020). The identification of more virulent strains of *S. capitis* ssp. *ureolyticus*, in greater frequencies in clinical settings combined with less virulent strains isolated from other sources, such as the scalp, could indicate a potential contextual basis for the HGT events. The addition of high-quality long-read genomic information from more extensive longitudinal studies, including sample collection from varying skin site locations, such as dry, moist, lipid-rich, and non-lipid-rich areas, as well as more clinical isolates, would allow further investigation into understanding the association between *S. capitis* subspecies and scalp skin state. Thus adding to the many studies using the higher resolution afforded by WGS, enabling important differences to be uncovered in *S. capitis* genomes, such as their multidrug resistance profiles across different geographic regions (Cui et al., 2013; Cameron et al., 2015; Carter et al., 2018).

Phylogenetic analysis revealed clustering of two distinct clades that likely represent the two subspecies, herein termed the *S. capitis* spp. *capitis* and the *S. capitis* spp. *ureolyticus* clade. Most of the clinical isolates from this study belonged to the *S. capitis* spp. *ureolyticus* clade and commensal isolates belonging to the *S. capitis* spp. *capitis* clade, suggesting that *S. capitis* spp. *ureolyticus* is more associated with clinical infections. This agrees with the current literature that indicates *S. capitis* spp. *ureolyticus* is the more virulent subspecies, which is linked to the presence of genes for biofilm formation and methicillin resistance (Cui et al., 2013; Tevell et al., 2020). The observation of multiple clinical isolates interspersed across the dominant subclade of *S. capitis* spp. *capitis* clade indicates that clinical and commensal isolates share a similar genetic background, and while *S. capitis* spp. *capitis* is less associated with a clinical infection, it can have disease potential.

Although the scalp-associated isolates sampled in this study were not from clinical infections, potential virulence-linked genes were found throughout their genomes, highlighting *S. capitis* versatility and potential for adaptation that might cause significant disease in settings like the NICU. Further exploration of sequence differences will be required to unravel defining features of *S. capitis* subspecies to support the hypothesis that *S. capitis* ssp. *ureolyticus*, or genomes belonging to the *S. capitis* ssp. *ureolyticus* clade, are generally more virulent.

Many published studies focus on *S. capitis* strains isolated from NICU and other clinical settings, describing the emergence of drug resistance in response to the use of antimicrobial and antiseptic therapy to treat CoNS infections (Mello et al., 2008; Rasigade et al., 2012; Chong et al., 2016; Carter et al., 2018; Wirth et al., 2020). Antimicrobial resistance to vancomycin and fusidic acid was reported among *S. capitis*, like *S. aureus*, suggesting the occurrence of inter-species genetic exchange (Chong et al., 2016; Carter et al., 2018).

In addition to antimicrobial resistance, biofilm formation was proposed to be an important virulence trait of *S. capitis* in both clinical and commensal settings (Otto, 2009; Cui et al., 2013). In keeping with this proposal, the current work confirms the presence of biofilm-related genes in the *S. capitis* genomes studied, including *icaRADBC* operon, *ebh* and *atlE*, and extends it by identifying an IcaC encoding gene cluster discriminating the subspecies with its presence in ssp. *ureolyticus*. The role of IcaC (Table 2) in *S. capitis* was described by Cui et al. as activity modifying synthesized glucan by acetylation (Cui et al., 2015). A more extensive investigation of IcaC, including *S. capitis* isolated from different sources, could contribute to further understanding trait differences that determine ssp. specialization. The suite of biofilm genes facilitates the primary attachment of staphylococci by binding to extracellular matrix molecules and intercellular aggregation (Otto, 2009). The presence of these genes may confer a selective advantage in both a clinical setting and on the scalp and forehead. Further phenotypic studies of biofilm formation, metabolism, and multidrug resistance in *S. capitis* isolates, including those in this study, will extend our knowledge of speciation and specialization of staphylococci and *S. capitis*, and could help with precise studies of factors that pertain to emergent clinical disease.

Currently, subdivision of *S. capitis* into ssp. *ureolyticus* and ssp. *capitis* is based upon original descriptions of *S. capitis* ssp. *ureolyticus* urease activity, ability to produce acid from maltose, fatty acid profile, larger colony size, and DNA sequence differentiation (Bannerman and Kloos, 1991). The multiple discriminating traits were not explored in full here, but biochemical analysis using the API *Staph-Ident* Strip system, the most common method to discriminate *S. capitis* subspecies, was tested on 14 isolates sampled from this study and 3 isolates from the culture collection type strains. The discrepancy between biochemical and whole-genome phylogenetic assignment of the subspecies was observed, as only 33% of the tested isolates that belonged to the *S. capitis* ssp. *ureolyticus* clade tested positive for urease activity, highlighting that classification of *S. capitis* subspecies by urease activity is unreliable and requires confirmation using other discriminating traits.

Further analysis to characterize the genetic relatedness between the two subspecies using ANI revealed that the genomes of each subspecies were similar, sharing 96% nucleotide identity. Instead, we investigated discriminating gene clusters and observed that *S. capitis* spp. *ureolyticus* genomes were enriched with antimicrobial resistance gene functions, such as β-lactam resistance genes and β-class phenol soluble modulins. However, *S. capitis* spp. *capitis* genomes were enriched with gene clusters linked to skin survival based on the presence of the arginine catabolism mobile element (ACME) that encodes enzymes to counteract low pH (Planet et al., 2013; Lindgren et al., 2014). ACME is a genomic island first described in *S. aureus* USA300 and in *S. epidermidis* ATCC 12228 (Diep et al., 2006; Planet et al., 2015). It was shown to enhance staphylococcal colonization of the skin and mucous membranes, showing similar characteristics to the staphylococcal cassette chromosome *mec* (SCC*mec*) element (Diep et al., 2008; Almebairik et al., 2020). While not investigated functionally here in *S. capitis*, it is likely to have a similar function. We hypothesize that ACME activity that discriminates ssp. *capitis* could represent a key factor of subspeciation through niche specialization. Analysis of virulence gene profiles determined that as a species, *S. capitis* has a similar repertoire of virulence genes to several other CoNS species, with respect to AMR, PSMs, and secreted proteases (Moawad et al., 2019; Sun et al., 2020). Investigation of the subspecies classifications highlighted here demonstrates that further analysis is required for robust markers of subspecies classification within their core genomes, although several genes exclusive to each subspecies were identified and could serve as subspecies biomarkers.

In conclusion, this study identified distinct clustering of the two subspecies of *S. capitis* and determined gene clusters for traits that might rapidly progress our understanding of *S. capitis* relevant to disease. Specifically, we propose that the original subspecies definition of ssp. *ureolyticus* needs to be reconsidered based on species subclades that define it based on the importance of MDR and virulence. It is likely that the widespread use of antimicrobials, the openness of the *S. capitis* pan-genome, and the acquisition of MGEs with beneficial mutations have promoted the emergence of virulence traits in *S. capitis* isolates. Continued research into the classification of *S. capitis* as subspecies vs. virulence-linked phylogroups will improve surveillance of the spread and evolution of *S. capitis*.

## Data availability statement

The datasets presented in this study can be found in online repositories. The names of the repository/repositories and accession number(s) can be found below: https://www.ebi.ac.uk/ena, PRJEB47273.

## Ethics statement

The studies involving human participants were reviewed and approved by an Independent Ethics Committee and operated at Unilever Port Sunlight, United Kingdom. The patients/participants provided their written informed consent to participate in this study.

## Author contributions

CC and MH conceived and designed this study and drafted the manuscript. CC performed all the data analysis and interpretation for the results under the scientific guidance of RB and MH. All authors contributed to the editing and approved the final manuscript.

## Funding

This work was funded by a Biotechnology and Biological Sciences Research Council Doctoral Training Partnership CASE studentship BB/M011186/1 awarded to MH, with support from Unilever PLC.

## Conflict of interest

The authors declare that the research was conducted in the absence of any commercial or financial relationships that could be construed as a potential conflict of interest.

## Publisher's note

All claims expressed in this article are solely those of the authors and do not necessarily represent those of their affiliated organizations, or those of the publisher, the editors and the reviewers. Any product that may be evaluated in this article, or claim that may be made by its manufacturer, is not guaranteed or endorsed by the publisher.

## Supplementary material

Supplementary Figure S1 Analysis of the *S. capitis* genome alignment with Gubbins. The maximum-likelihood phylogenetic reconstruction of *S. capitis* is shown on the left, with colored bands highlighting rhierBAPS clustering and isolation site of genomes. Filled gray triangles describe scalp isolates from this study. Homologous recombination events for each *S. capitis* genome ordered based on their position in the AYP1020 reference genome (shown along the top) are shown on the right. Recombination blocks detected in >1 isolate are shown in red, while blocks affecting a single isolate are indicated in blue. Figure is visualized using Phandango (Hadfield et al., 2017).

Supplementary Figure S2 *Staphylococcus capitis* API-Staph test results. Representative *S. capitis* ssp. *capitis* and ssp. *ureolyticus* isolates, including culture collection type strains, were biochemically analyzed to confirm phenotypic signature of subspecies. ML tree is midpoint rooted, and bootstrap support values were calculated from 1,000 replicates. The first color block represents rhierBAPS clustering, and dots describe the setting where isolates were retrieved; green = commensal (including scalp samples from this study), red = clinical, and gray = unknown. Filled gray triangles describe scalp isolates from this study. The subspecies differentiation of *S. capitis* is presented as the subclades described as BAPS groups 1, 3, 4, and 5. Tests include substrates (from left to right): NO substrate, D-GLUcose, D-FRUctose, D-ManNosE, D-MALtose, D-LACtose, D-TREhalose, D-MANnitol, XyLiTol, D-MELibiose, potassium NITrate, β-naphthly phosphate, sodium pyruvate, D-RAFfinose, D-XYLose, D-SACcharose, Methy-αD-Glucopyranoside, N-Acetyl-Glucosamine, L-arginine, and UREa.

Supplementary Table S1 *Staphylococcus capitis* isolates used in this study. Isolates were either isolated in this study, type strains, or sequence data obtained from the NCBI database.

Supplementary Table S2 Genes significantly differentiating *S. capitis* subspecies. Gene identification output from Scoary analysis.

Supplementary Table S3 Representative protein sequences of hypothetical gene clusters that significantly differentiate *S. capitis* subspecies. Gene identification output from Scoary analysis and protein sequence information from annotated genome assemblies.
