# Supplementary figures and images for: Comparative genomics of Staphylococcus capitis reveals species determinants
Source: Front Microbiol. 2022 Sep 30;13:1005949. doi: 10.3389/fmicb.2022.1005949 (PMC9563023; doi:10.3389/fmicb.2022.1005949)

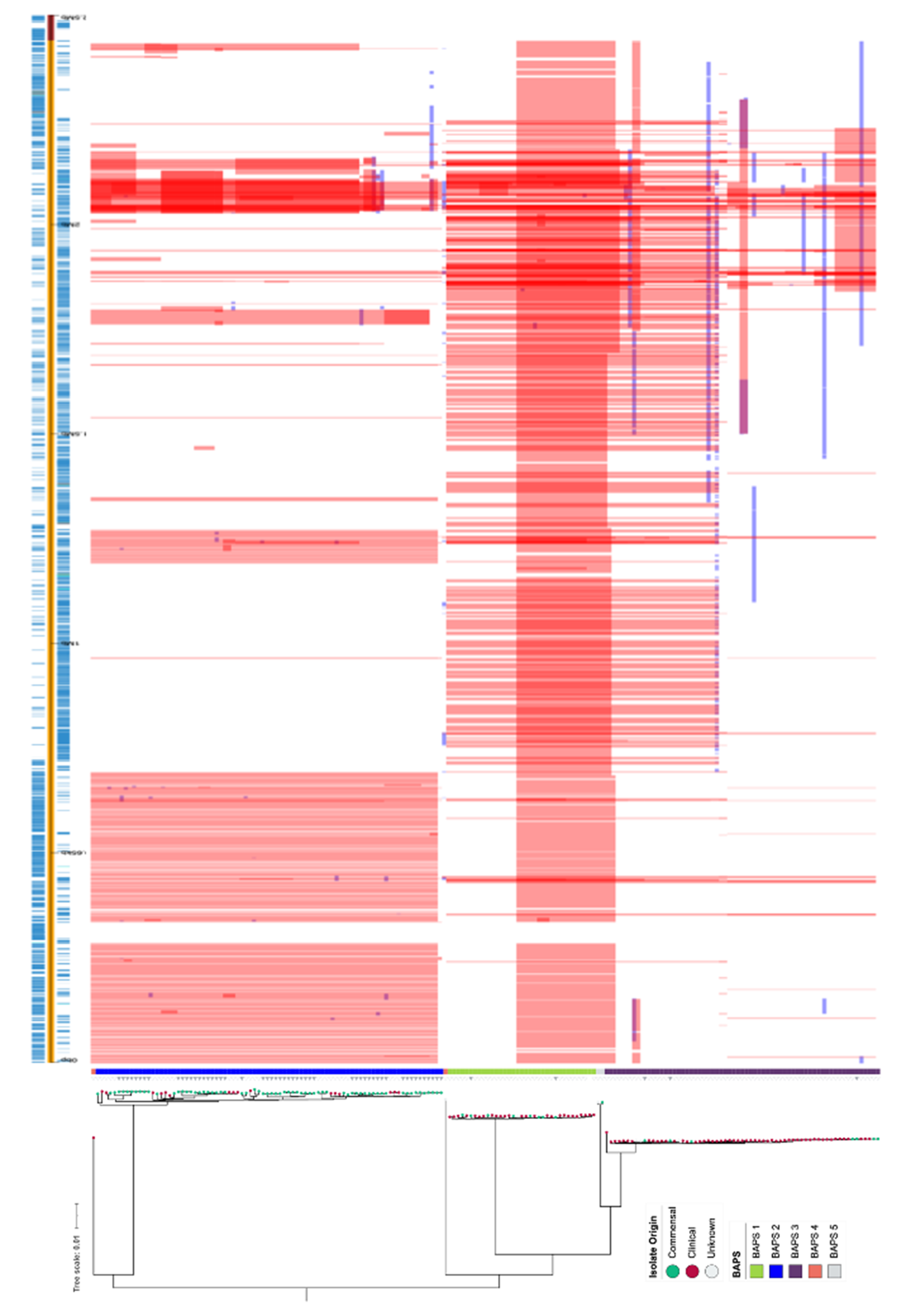

Supplement: Supplementary Figure S1 — Analysis of the S. capitis genome alignment with Gubbins. The maximum-likelihood phylogenetic reconstruction of S. capitis is shown on the left, with colored bands highlighting rhierBAPS clustering and isolation site of genomes. Filled gray triangles describe scalp isolates from this study. Homologous recombination events for each S. capitis genome ordered based on their position in the AYP1020 reference genome (shown along the top) are shown on the right. Recombination blocks detected in >1 isolate are shown in red, while blocks affecting a single isolate are indicated in blue. Figure is visualized using Phandango (Hadfield et al., 2017). [file Image_1.TIFF]

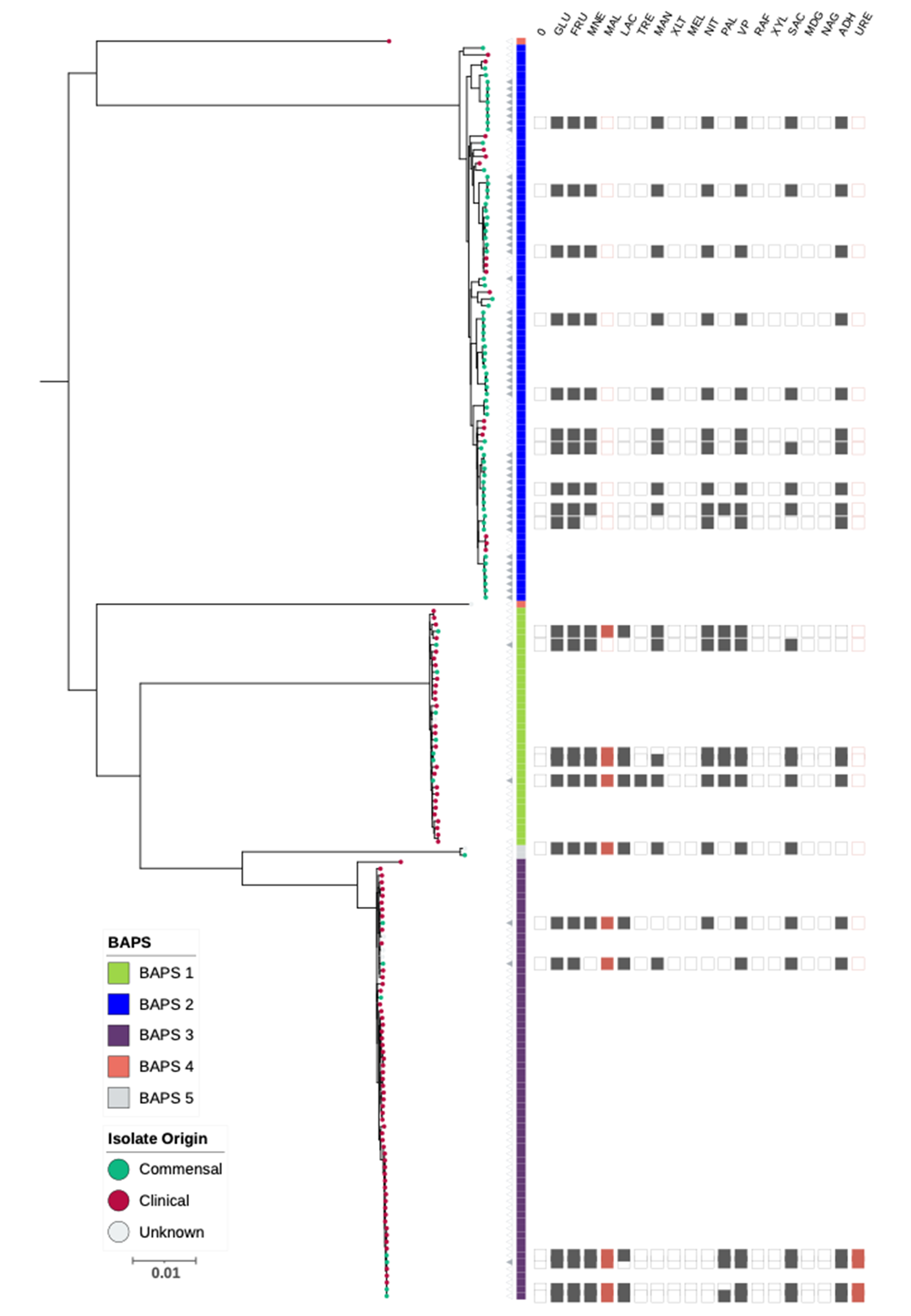

Supplement: Supplementary Figure S2 — Staphylococcus capitis API-Staph test results. Representative S. capitis ssp. capitis and ssp. ureolyticus isolates, including culture collection type strains, were biochemically analyzed to confirm phenotypic signature of subspecies. ML tree is midpoint rooted, and bootstrap support values were calculated from 1,000 replicates. The first color block represents rhierBAPS clustering, and dots describe the setting where isolates were retrieved; green = commensal (including scalp samples from this study), red = clinical, and gray = unknown. Filled gray triangles describe scalp isolates from this study. The subspecies differentiation of S. capitis is presented as the subclades described as BAPS groups 1, 3, 4, and 5. Tests include substrates (from left to right): NO substrate, D-GLUcose, D-FRUctose, D-ManNosE, D-MALtose, D-LACtose, D-TREhalose, D-MANnitol, XyLiTol, D-MELibiose, potassium NITrate, β-naphthly phosphate, sodium pyruvate, D-RAFfinose, D-XYLose, D-SACcharose, Methy-αD-Glucopyranoside, N-Acetyl-Glucosamine, L-arginine, and UREa. [file Image_2.TIFF]
